# A Positive Correlation between Atypical Memory B Cells and *Plasmodium falciparum* Transmission Intensity in Cross-Sectional Studies in Peru and Mali

**DOI:** 10.1371/journal.pone.0015983

**Published:** 2011-01-14

**Authors:** Greta E. Weiss, Eva H. Clark, Shanping Li, Boubacar Traore, Kassoum Kayentao, Aissata Ongoiba, Jean N. Hernandez, Ogobara K. Doumbo, Susan K. Pierce, OraLee H. Branch, Peter D. Crompton

**Affiliations:** 1 Laboratory of Immunogenetics, National Institute of Allergy and Infectious Diseases, National Institutes of Health, Rockville, Maryland, United States of America; 2 Department of Microbiology, University of Alabama at Birmingham, Birmingham, Alabama, United States of America; 3 Laboratorio de Investigación de Productos Naturales Antiparasitarios de la Amazonia (LIPNAA), Universidad Nacional de la Amazonia Peruana, Iquitos, Loreto, Peru; 4 Malaria Research and Training Centre, Department of Epidemiology of Parasitic Diseases, Faculty of Medicine, Pharmacy, and Odonto-Stomatology, University of Bamako, Bamako, Mali; 5 Department of Medical Parasitology, New York University, New York, New York, United States of America; The George Washington University Medical Center, United States of America

## Abstract

**Background:**

Antibodies that protect against *Plasmodium falciparum* (*Pf*) malaria are only acquired after years of repeated infections. The B cell biology that underlies this observation is poorly understood. We previously reported that “atypical” memory B cells are increased in children and adults exposed to intense *Pf* transmission in Mali, similar to what has been observed in individuals infected with HIV. In this study we examined B cell subsets of *Pf* -infected adults in Peru and Mali to determine if *Pf* transmission intensity correlates with atypical memory B cell expansion.

**Methodology/Principal Findings:**

In this cross-sectional study venous blood was collected from adults in areas of zero (U.S., n = 10), low (Peru, n = 18) and high (Mali, n = 12) *Pf* transmission. Adults in Peru and Mali were infected with *Pf* at the time of blood collection. Thawed lymphocytes were analyzed by flow cytometry to quantify B cell subsets, including atypical memory B cells, defined by the cell surface markers CD19^+^ CD20^+^ CD21^−^ CD27^−^ CD10^−^. In Peru, the mean level of atypical memory B cells, as a percent of total B cells, was higher than U.S. adults (Peru mean: 5.4% [95% CI: 3.61–7.28]; U.S. mean: 1.4% [95% CI: 0.92–1.81]; p<0.0001) but lower than Malian adults (Mali mean 13.1% [95% CI: 10.68–15.57]; p = 0.0001). In Peru, individuals self-reporting ≥1 prior malaria episodes had a higher percentage of atypical memory B cells compared to those reporting no prior episodes (≥1 prior episodes mean: 6.6% [95% CI: 4.09–9.11]; no prior episodes mean: 3.1% [95% CI: 1.52–4.73]; p = 0.028).

**Conclusions/Significance:**

Compared to *Pf*-naive controls, atypical memory B cells were increased in Peruvian adults exposed to low *Pf* transmission, and further increased in Malian adults exposed to intense *Pf* transmission. Understanding the origin, function and antigen specificity of atypical memory B cells in the context of *Pf* infection could contribute to our understanding of naturally-acquired malaria immunity.

## Introduction

Passive transfer studies in humans indicate that antibodies (Abs) play a critical role in controlling the disease associated with the asexual blood stages of *Plasmodium falciparum* (*Pf*) malaria [1], [2]. However, Abs that protect against clinical malaria are only acquired after repeated infections and may be relatively short-lived [3], [4]. This stands in contrast to the long-term or even lifelong Ab-mediated immunity that follows one or a few exposures to many viral and bacterial antigens, either through natural exposure or vaccination [5].

Recent advances in B cell biology have now established that long-term protective humoral immunity requires the generation and maintenance of memory B cells (MBCs) and long-lived plasma cells (LLPCs), defined by the cell surface markers CD19+CD27+CD38− and CD19+CD27++CD38+++, respectively (reviewed in [6], [7], [8]). The process of generating MBCs and LLPCs begins when naïve B cells encounter their cognate antigen near the interface of B and T cell areas of secondary lymphoid tissue. Several studies suggest that high-affinity antigen binding drives naïve B cells to differentiate into short-lived, isotype-switched plasma cells (PCs) within the extra-follicular region which contributes to the initial control of infections. In contrast, lower affinity binding selects for entry of naïve B cells into follicles where germinal centers are formed. After a period of 7–10 days, during which the CD4^+^ T-cell-dependent process of affinity maturation and immunoglobulin class-switching occurs, the germinal center reaction yields LLPCs and MBCs of higher affinity than the initial wave of short-lived plasma cells (SLPCs). LLPCs migrate to the bone marrow where they constitutively secrete antibody and provide a critical first line of defense against re-infection, whereas MBCs recirculate and mediate recall antibody responses after re-exposure to their cognate antigen by rapidly proliferating and differentiating into PCs.

The MBC and LLPC response following exposure to many pathogens and vaccines is remarkably efficient and durable [5], [9]. For example, the smallpox vaccine reliably induces vaccine-specific MBCs and long-lived Abs that persist for ≥50 years in the absence of antigen re-exposure [9]. By comparison, the MBC and LLPC response to *Pf* infection appears to be less efficient. Recent studies in areas of high and low *Pf* transmission have shown that MBCs specific for certain *Pf* blood stage antigens are detectable in only 30–50% of adults [10], [11], [12]. Furthermore, the Ab response to many *Pf* antigens following natural infection appears to be dominated by SLPCs rather than LLPCs [4], [13]. Although antigenic variation likely contributes to the delayed acquisition of antibodies that protect against clinical malaria [14], the mechanisms underlying what appears to be a relatively inefficient B cell response to *Pf* infection remain poorly defined.

We recently reported that *Pf* exposure in Malian children and adults is associated with the expansion of a phenotypically distinct population of MBCs identified by the cell surface markers CD19^+^ CD20^+^ CD21^−^ CD27^−^ CD10^−^ [15], a B cell subpopulation that was initially defined by expression of the inhibitory receptor Fc-receptor-like-4 (FCRL4) [16]. This observation provided a clue that *Pf* may modulate the humoral immune response at the level of MBCs. B cells with a similar phenotype have also been identified in individuals infected with HIV [17] and HCV [18]. Moir et al. showed that this subset of B cells in HIV-infected individuals had undergone isotype class switching and somatic hypermutation, but compared to naive B cells and classical MBCs, FCRL4^+^ MBCs proliferated less well to BCR-crosslinking and/or the CD40L and Toll-like receptor 9 (TLR9) agonist CpG, and showed a decreased ability to differentiate into antibody secreting cells in response to CpG and the polyclonal activator *Staphylococcus aureus* Cowan (SAC) [17]. FCRL4^+^ MBCs in HIV-viremic [17] and *Pf*-exposed individuals [15] also express high levels of inhibitory receptors and a profile of lymphoid-homing receptors similar to what is expressed on exhausted CD8^+^ T cells during chronic viral infections (12). Although the function of FCRL4^+^ MBCs remains unknown in the context of HIV and malaria, Moir *et al*. suggested that this subset of hyporesponsive, or ‘exhausted’, FCRL4^+^ MBCs contribute to the B cell deficiencies observed in HIV-infected individuals [17]. In contrast, Ehrhardt *et al*. [16], who first described FCRL4^+^ ‘tissue-like’ MBCs in lymphoid tissues of healthy individuals, suggested that these cells may play a protective role during infection. Given that the function of FCRL4^+^ MBCs in *Pf*-exposed individuals is unknown, we refer to this B cell subset in the context of malaria as ‘atypical’ rather than ‘exhausted’.

To gain further insight into the relationship between atypical MBCs and *Pf* infection we examined by flow cytometry the B cell subsets of *Pf* -infected adults in Peru and Mali, and *Pf*-naive adults in the U.S. The specific objectives of this study were to determine if atypical MBC expansion is an observation that can be generalized to *Pf*-exposed individuals in Peru, and if there is a correlation between the degree of atypical MBC expansion and *Pf* transmission intensity. We observed that atypical MBCs were expanded in *Pf*-infected individuals in Peru relative to *Pf*-naive individuals, and that the degree of atypical MBC expansion increased with increasing *Pf* transmission intensity.

## Results

### Study subject characteristics

In Peru, PBMCs from 18 adults with symptomatic *Pf* infection were analyzed. The mean parasite density was 10,226 asexual parasites/µl of blood [95% CI: 2,618–17,833]). The average age of study participants was 40.4 years (range: 20–75 years) and 50% were female. All 18 adults reported subjective fever, chills and other symptoms consistent with malaria in the two days prior to enrolment. At the time of presentation to the study clinic, 6 of 18 participants had a temperature ≥37.5°C, and 12 self-reported at least one prior episode of symptomatic malaria diagnosed by blood smear. Of note, community-wide active surveillance surveys at this site indicate that up to 40% of *Pf* infections in adults are asymptomatic [19], despite the low intensity of *Pf* transmission. A detailed description of the study site and design in Peru has been reported elsewhere [19].

In Mali, PBMCs from 12 adults with asymptomatic *Pf* infection were analyzed. The mean parasite density was 410 asexual parasites/µl of blood [95% CI: -187-1008]). The average age of study participants was 21.1 years (range: 19–25 years) and 50% were female. At the time of presentation to the study clinic, all subjects were afebrile. A detailed description of the study site and design in Mali has been reported elsewhere [20].

### Atypical MBC analysis

**Figure 1:**
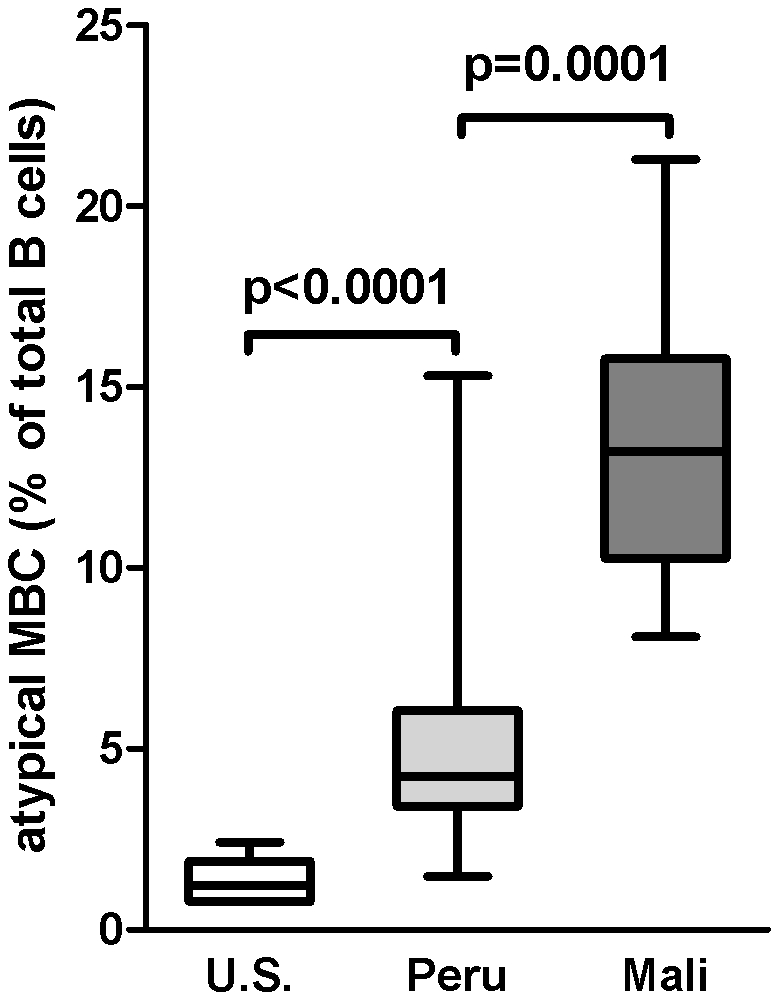
Atypical MBCs in individuals from the U.S., Peru and Mali. Atypical MBCs were detected by flow cytometry and expressed as a percentage of total B cells in the peripheral blood of *Pf*-naive U.S. adults (n = 10), *Pf*-infected Peruvian adults (n = 18) and *Pf*-infected Malian adults (n = 12). Box-and-whisker plots represent the smallest and largest values (whiskers), the lower and upper quartiles (top and bottom of box), and the median (horizontal line across box). The Mann-Whitney test was used to compare continuous variables between groups.

**Figure 2:**
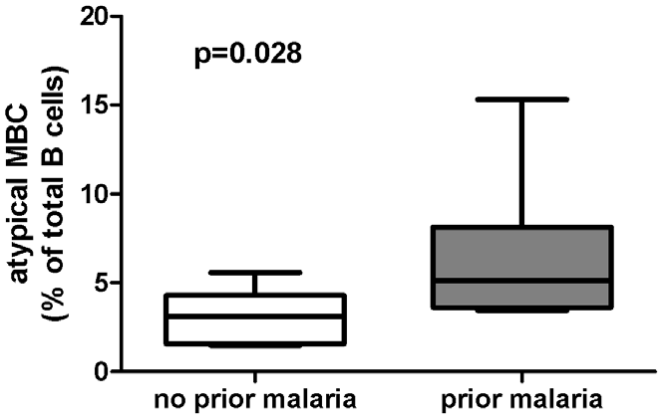
Atypical MBCs in Peruvian adults by self-reported history of prior malaria episodes. Atypical MBCs were detected by flow cytometry and expressed as a percentage of total B cells in the peripheral blood of *Pf*-infected Peruvian adults who did (n = 12) or did not (n = 6) self-report a prior history of malaria. Box-and-whisker plots represent the smallest and largest values (whiskers), the lower and upper quartiles (top and bottom of box), and the median (horizontal line across box). The Mann-Whitney test was used to compare continuous variables between groups.

We determined if atypical MBCs were detectable in the peripheral blood of Peruvian adults (n = 18) living in an area of low level *Pf* transmission by performing FACS analysis on thawed PBMCs which had been collected during symptomatic *Pf* infection. These data were compared to B cell profiles of *Pf*-naive U.S. adults (n = 10) and *Pf*-infected asymptomatic Malian adults (n = 12). As a percentage of total B cells, the mean percentage of atypical MBCs in Peruvian adults was higher than the mean percentage in U.S. adults (Fig. 1; Peru: 5.4% [95% CI: 3.6–7.3]; U.S.: 1.4% [95% CI: 0.9–1.8; P<0.0001], but lower than the percentage in Malian adults (Fig. 1; Mali: 13.1% [95% CI: 10.7–15.6]; P = 0.0001 versus Peru]. The low percentage of atypical MBCs in the peripheral blood of healthy U.S. adults is consistent with other reports [17]. Stratifying Peruvian adults by a self-reported history of prior malaria episodes revealed that the mean percentage of atypical MBCs was higher in those reporting at least one prior malaria episode (n = 12), versus those who reported no prior malaria episodes (n = 6) (Fig. 2; no prior malaria reported: 3.1% [95% CI: 1.5–4.7]; prior malaria reported: 6.6% [95% CI: 4.1–9.1; P = 0.028]. Together these data suggest that atypical MBCs increase as a percentage of total B cells with increasing *Pf* exposure.

### Phenotypic analysis of other B cell subsets

**Figure 3:**
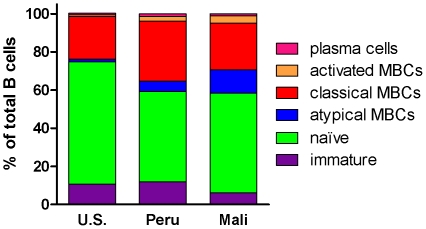
Phenotypic analysis of B cell subsets in individuals from the U.S., Peru and Mali. The following B cell subsets were quantified by flow cytometry, as detailed in *Materials and Methods*: plasma cells/blasts (CD19^+^ CD21^−^ CD20^−^), activated MBCs (CD19^+^ CD21^−^ CD27^+^CD20^+^), classical MBCs (CD19^+^ CD27^+^ CD21^+^), atypical MBCs (CD19^+^ CD20^+^ CD21^−^ CD27^−^ CD10^−^) naive B cells and immature B cells (CD19^+^ CD10^+^). B cell subsets are expressed as a percentage of total CD19^+^ B cells in the peripheral blood of *Pf*-naive U.S. adults (n = 10), *Pf*-infected Peruvian adults (n = 18) and *Pf*-infected Malian adults (n = 12). The relative proportions of B cell subsets are shown in stacked plots.

In addition to the variation we observed in the proportion of atypical MBCs between study populations in the U.S., Peru, and Mali, we found significant differences in the proportions of other B cell subsets (Fig. 3). The proportion of activated MBCs was higher in Peru (n = 12) and Mali (n = 13) compared to the U.S. (n = 10) (Peru: 2.5% [95% CI: 1.4–3.6], Mali: 4.0% [95% CI: 3.2–4.8], U.S.: 0.9% [95% CI: 0.3–1.6]; Peru vs. U.S. p = 0.003; Mali vs. U.S. p = 0.0003), consistent with concurrent *Pf* infection in donors from Peru and Mali. In addition, the proportion of naïve B cells was lower in Peru and Mali compared to the U.S. (Peru: 47.6% [95% CI: 41.2–54.1], Mali: 54.3% [95% CI: 48.6–59.9], U.S.: 64.2% [95% CI: 56.3–72.0]; Peru vs. U.S. p = 0.003; Mali vs. U.S. p = 0.013); and the proportion of classical MBCs was higher in Peru and Mali compared to the U.S. (Peru: 31.6% [95% CI: 26.5–36.7], Mali: 23.3% [95% CI: 18.0–28.6], U.S.: 22.4% [95% CI: 16.6–28.2]; Peru vs. U.S. p = 0.04; Mali vs. U.S. p = 0.82), although the difference between the U.S. in Mali did not reach statistical significance. The decrease in naïve B cells and increase in classical MBCs in Peruvians and Malians relative to the U.S. donors may reflect greater cumulative immunological experience of study participants in Peru and Mali.

### Atypical MBCs and immunoglobulin class switching

**Figure 4:**
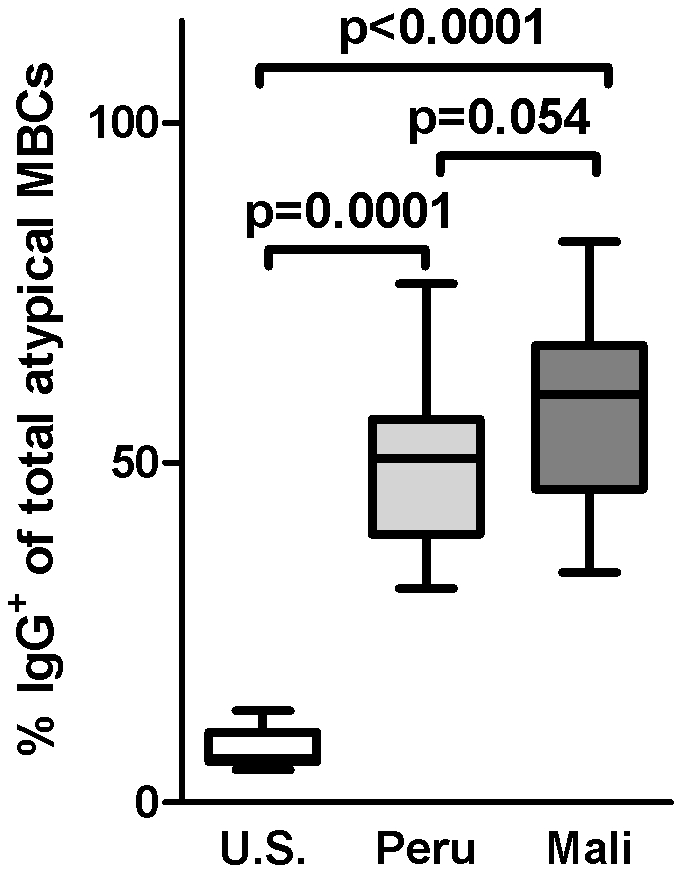
Atypical MBCs and immunoglobulin class switching. Class-switching of atypical MBCs was assessed by measuring cell surface expression of IgG by flow cytometry in the peripheral blood of *Pf*-naive U.S. adults (n = 10), *Pf*-infected Peruvian adults (n = 18) and *Pf*-infected Malian adults (n = 12). Box-and-whisker plots represent the smallest and largest values (whiskers), the lower and upper quartiles (top and bottom of box), and the median (horizontal line across box). The Mann-Whitney test was used to compare continuous variables between groups.

Immunoglobulin class switching occurs in germinal center reactions during the differentiation of naïve B cells into MBCs. We assessed class-switching of atypical MBCs by measuring cell surface expression of IgG by flow cytometry (Fig. 4). The mean percentage of class-switched atypical MBCs was significantly higher among individuals in Peru and Mali compared to the U.S. (Peru: 48.0% [95%CI: 41.1–54.9], Mali: 55.1% [95%CI: 43.3–66.8], U.S.: 8.1% [95% CI: 5.9–10.3]; Peru vs. U.S., p = 0.0001; Mali vs. U.S., p<0.0001). The higher percentage of class switched atypical MBCs in individuals from Mali versus individuals from Peru was not statistically significant (p = 0.054). The percentage difference in class switched atypical MBCs between individuals in Peru who did, and did not self-report prior malaria was not statistically significant (Peru, no prior malaria: 44.8% [95% CI: 28.58–60.98], Peru, prior malaria: 49.6% [95% CI: 41.12–58.10]; p = 0.64).

## Discussion

We reported previously that atypical MBCs, as a percentage of total B cells, are expanded in children and adults exposed to intense seasonal *Pf* transmission in Mali [15]. The objectives of the present study were to determine if atypical MBC expansion is a finding that can be generalized to *Pf*-exposed individuals in Peru, and if there is a correlation between the degree of atypical MBC expansion and *Pf* transmission intensity. We observed that atypical MBCs were expanded in *Pf*-infected individuals in Peru relative to *Pf*-naive individuals, and that the degree of atypical MBC expansion increased with increasing *Pf* transmission intensity, consistent with the hypothesis that *Pf* infection drives the expansion of atypical MBCs.

Although this study further strengthens the association between *Pf* infection and atypical MBCs, a causal relationship remains to be established. Many factors associated with *Pf* transmission could account for the increase in atypical MBCs observed in Peru and Mali, including concurrent helminth infections, cumulative exposure to other pathogenic and non-pathogenic microbes, and malnutrition. It will be important to examine the role of these and other factors in the expansion of atypical MBCs in future studies. Although we previously reported a lack of association between concurrent intestinal helminth infection and the degree of atypical MBC expansion [15], this observation needs to be confirmed in other studies. It is unlikely that HIV drives the atypical MBC response observed in the present study since the prevalence of HIV is <1.5% in the areas of Mali [21] and Peru (O. Branch, unpublished data) where this study was conducted. It also seems less likely that host genetic factors play a role in atypical MBC expansion since this study suggests that atypical MBCs appear to be a generalizable finding in *Pf*-exposed individuals of different ethnic backgrounds on separate continents.

From an epidemiological standpoint, additional studies are needed to further test the hypothesis that *Pf* infection drives the atypical MBC response. For example, comparing B cell subsets of genetically related individuals living under similar conditions with the exception of *Pf* exposure would help isolate the effect of *Pf* infection on atypical MBC expansion. Tracking B cell profiles longitudinally in individuals residing in areas where *Pf* transmission is rapidly decreasing [22], or in individuals emigrating from *Pf* endemic areas could also provide insights into the temporal relationship between *Pf* exposure and the longevity of atypical MBCs. Additionally, B cell profiles of children in the treatment and control arms of trials of intermittent preventive treatment (IPTc) of malaria [23] could be compared to determine if and how decreased *Pf* exposure influences the atypical MBC response.

The specificity and function of atypical MBCs in the context of *Pf* infection remains to be determined. In the case of HIV, Moir et al found that HIV-specific MBCs were enriched in ‘exhausted’ MBCs whereas the total immunoglobulin and influenza-specific responses were enriched in classical MBCs [17]. Further studies are needed to determine the degree to which atypical MBCs are *Pf*-specific. Obstacles in this regard are the relatively low frequency of MBCs of any given antigen specificity in the peripheral circulation and the small volumes of blood typically collected in field studies. The latter could be overcome by establishing safe and reliable leukopheresis facilities where such studies are done.

Regarding the function of atypical MBCs, Ehrhardt *et al,* who first described FCRL4^+^ ‘tissue-like’ MBCs in the lymphoid tissue of healthy individuals [16], suggested that these cells may protect against invasive pathogens possibly through their influence on other cells, either directly or indirectly through the secretion of cytokines [24], [25]. In contrast, Moir *et al*. suggested that this subset of hyporesponsive, or ‘exhausted’ FCRL4^+^ MBCs contribute to the B cell deficiencies observed in HIV-infected individuals [17]. By analogy, it is possible that atypical MBCs confer protection against malaria by contributing to the regulation of the host immune response; on the other hand, it is also conceivable that exhaustion of *Pf*-specific MBCs through repeated infections contributes to the inefficient acquisition and relatively rapid loss of *Pf*-specific MBCs and long-lived antibodies that we have observed in the same study population in Mali [12], [13]. Immunoglobulin class switching and somatic hypermutation occur simultaneously in germinal center reactions during the differentiation of naïve B cells into MBCs. More than half of the atypical MBCs detected in individuals in Peru and Mali expressed surface IgG, indicating that at least a portion of these cells have passed through a germinal center reaction. This is consistent with models in which atypical MBCs are either the product of a dysfunctional/aborted germinal center reaction, or arise from pre-existing classical MBCs; either scenario could lead to a diminished classical MBC response.

In summary, this study provides evidence that atypical MBC expansion is a generalizable finding in *Pf*-exposed populations, and that the magnitude of the atypical MBC response increases with increasing *Pf* transmission intensity. Further studies are needed to define the origin, function and antigen specificity of atypical MBCs in the context of malaria.

## Materials and Methods

### Peru study site and participants

The Malaria Immunology and Genetics in the Amazon (MIGIA) study began in 2003 and is ongoing in an area just south of Iquitos, Peru, as detailed elsewhere [26]. *Pf* and *Plasmodium vivax (Pv)* transmission at this site occurs during the rainy season from January through July. Although entomological inoculation rate (EIR) data is not available at this site, the incidence of symptomatic *Pf* and *Pv* infection was low in 2003—approximately 0.03 and 0.22 infections/person/malaria season, respectively [26]. The period prevalence of asymptomatic *Pf* and *Pv* infection by blood smear during one month at the beginning of the malaria season in 2003 was approximately 12.5% and 37.8%, respectively [26]. The present cross-sectional study includes 18 adults who presented to the study clinic with symptomatic *Pf* infection during the 2009 malaria season. At enrolment a physician examined the study participants and solicited a medical history which included the self-reported number of prior malaria episodes. A finger-prick blood sample was obtained for a blood smear and 8ml of venous blood were drawn for analysis by flow cytometry. After the blood draw, malaria treatment was provided following international standards. The Ethical Review Board of the Peruvian National Institute of Health and Ministry of Health, and the Institutional Review Boards (IRB) of New York University and the University of Alabama approved this study. Written, informed consent was obtained from all study participants.

### Mali study site and participants

In May 2006 an observational cohort study of naturally-acquired malaria immunity was initiated in Kambila, Mali, a rural village with a population of 1500 located 20 km north of the capital of Bamako. A detailed description of the study site and cohort design are reported elsewhere [27]. *Pf* transmission is seasonal and intense at this site from July through December. The entomological inoculation rate measured in a nearby village was approximately 50–60 infective bites/person/month in October 2000 and fell to near zero during the dry season [28]. The present cross-sectional study includes 12 adults enrolled in the cohort study who had a blood smear positive for *Pf* at a scheduled follow-up visit during the 2006 malaria season, and who had an available sample of frozen peripheral blood mononuclear cells (PBMCs) that had been drawn during the same visit. As is typical in Mali, these *Pf*-infected adults had no signs or symptoms of malaria when the blood smear was prepared and they were not treated with anti-malarial drugs (the blood smear was not read immediately). The Ethics Committee of the Faculty of Medicine, Pharmacy, and Odonto-Stomatology, and the IRB at the National Institute of Allergy and Infectious Diseases (NIAID), National Institutes of Health (NIH) approved this study (protocol # 06-I-N147). Written, informed consent was obtained from all study participants.

### U.S. blood donors

PBMCs from ten healthy adult blood bank donors in the U.S. were also analyzed. Demographic and travel history data were not available from these anonymous donors but prior *Pf* exposure is unlikely. Blood samples were obtained for research use after written informed consent was obtained from all study participants enrolled in a protocol approved by the IRB of NIAID, NIH (protocol # 99-CC-0168).

### PBMC collection

In Peru, Mali, and the U.S., blood samples were drawn by venipuncture into sodium citrate-containing cell preparation tubes (BD, Vacutainer CPT Tubes). PBMCs were isolated according to the manufacturer's instructions and frozen in fetal bovine serum (FBS) (Gibco, Grand Island, NY) containing 7.5% dimethyl sulfoxide (DMSO; Sigma-Aldrich, St. Louis, MO). PBMCs were kept at −80°C for 24 hours and then transferred to −196°C in the U.S. and Mali, or −135°C in Peru. We verified previously that the expansion of atypical MBCs is not an artifact of freezing and thawing PBMCs [15].

### Phenotypic analysis of B cell subsets

All phenotypic analyses were performed using mouse monoclonal antibodies specific for human B cell markers conjugated to fluorophores as previously reported [29]. Fluorophore-conjugated monoclonal antibodies specific for the following markers were used: PECy7-CD19, PE-CD20, APC-CD10, APC-CD27, PE-IgG (BD Biosciences, San Jose, CA) and FITC-CD21 (Beckman Coulter, Fullerton, CA). A four-color, two-stain strategy was used to identify B cell subsets as follows: plasma cells/blasts (CD19^+^ CD21^−^ CD20^−^), immature B cells (CD19^+^ CD10^+^), classical MBCs (CD19^+^ CD27^+^ CD21^+^), atypical MBCs (CD19^+^ CD20^+^ CD21^−^ CD27^−^ CD10^−^and activated MBCs (CD19^+^ CD21^−^ CD27^+^CD20^+^). As we and Moir et al previously reported, FCRL4 is primarily expressed on the CD19^+^ CD20^+^ CD21^−^ CD27^−^ CD10^−^ B cell subset; however, levels of FCRL4 expression are variable whereas CD20 clearly and consistently delineates the atypical memory B cells [15], [17]. FACS analyses were performed on a FACSCalibur flow cytometer (BD Biosciences) using FlowJo software (Tree Star, Ashland, OR).

### Measurement of peripheral blood *Pf* parasitemia

In Mali and Peru, thick blood smears were stained with Giemsa and counted against 300 leukocytes. *Pf* densities were recorded as the number of asexual parasites/µl of whole blood, based on an average leukocyte count of 7500/µl in Mali and 6000/µl in Peru. Each smear was evaluated separately by two expert microscopists blinded to the clinical status of study participants. Any discrepancies were resolved by a third expert microscopist.

### Statistical analysis

Data were analyzed using GraphPad Prism for Windows (GraphPad Software, version 5.02).The non-parametric Mann-Whitney test was used to compare continuous variables between groups, and the Fisher's exact test was used to compare categorical variables. For all tests, two-tailed p values were considered significant if ≤0.05.
